# Factors affecting brain structure in smoking-related diseases: Chronic Obstructive Pulmonary Disease (COPD) and coronary artery disease

**DOI:** 10.1371/journal.pone.0259375

**Published:** 2021-11-05

**Authors:** Catherine A. Spilling, Mohani-Preet K. Dhillon, Daniel R. Burrage, Sachelle Ruickbie, Emma H. Baker, Thomas R. Barrick, Paul W. Jones

**Affiliations:** 1 Neurosciences Research Centre, Molecular and Clinical Sciences Research Institute, St George’s University of London, London, United Kingdom; 2 Institute for Infection and Immunity, St George’s University of London, London, United Kingdom; 3 Respiratory Medicine, St George’s University Hospitals NHS Foundation Trust, London, United Kingdom; University at Buffalo, UNITED STATES

## Abstract

**Background:**

Changes in brain structure and cognitive decline occur in Chronic Obstructive Pulmonary Disease (COPD). They also occur with smoking and coronary artery disease (CAD), but it is unclear whether a common mechanism is responsible.

**Methods:**

Brain MRI markers of brain structure were tested for association with disease markers in other organs. Where possible, principal component analysis (PCA) was used to group markers within organ systems into composite markers. Univariate relationships between brain structure and the disease markers were explored using hierarchical regression and then entered into multivariable regression models.

**Results:**

100 participants were studied (53 COPD, 47 CAD). PCA identified two brain components: brain tissue volumes and white matter microstructure, and six components from other organ systems: respiratory function, plasma lipids, blood pressure, glucose dysregulation, retinal vessel calibre and retinal vessel tortuosity. Several markers could not be grouped into components and were analysed as single variables, these included brain white matter hyperintense lesion (WMH) volume. Multivariable regression models showed that less well organised white matter microstructure was associated with lower respiratory function (*p* = 0.028); WMH volume was associated with higher blood pressure (*p* = 0.036) and higher C-Reactive Protein (*p* = 0.011) and lower brain tissue volume was associated with lower cerebral blood flow (*p*<0.001) and higher blood pressure (*p =* 0.001). Smoking history was not an independent correlate of any brain marker.

**Conclusions:**

Measures of brain structure were associated with a range of markers of disease, some of which appeared to be common to both COPD and CAD. No single common pathway was identified, but the findings suggest that brain changes associated with smoking-related diseases may be due to vascular, respiratory, and inflammatory changes.

## Introduction

Chronic Obstructive Pulmonary Disease (COPD) and coronary artery disease (CAD) are both associated with cognitive impairment and changes to brain structure [1–3], but the mechanisms have not been identified. Smoking, and cigarette smokers have an elevated risk of cognitive decline, cerebrovascular disease and dementia [4, 5]. Meta-analyses have shown that current smoking increases the risk of Alzheimer’s disease and vascular dementia compared to never smoking [5]. Relationships have also been found between greater pack year smoking history and greater rate of cognitive decline [6].

COPD and CAD frequently co-occur and, when combined, are associated with worse prognosis than either disease in isolation [7]. The two diseases are also linked. Cardiovascular disease increases the risk of exacerbations [8] and is a major cause of mortality in COPD [9]. COPD increases the risk of angina, myocardial infarction, hospitalisation and cardiovascular mortality [10, 11], with patients being particularly vulnerable to acute vascular events during the peri-exacerbation period [12]. The two diseases also share a number of risk factors including smoking, ageing, sedentary lifestyle and exposure to air pollution, although these risk factors do not fully explain their co-occurrence [13].

Magnetic resonance imaging (MRI) studies show that smoking is associated with progression of white matter hyperintense lesions (WMHs) of presumed vascular origin [14] and both total and localised reduction in grey matter volume [15, 16]. Diffusion tensor imaging (DTI) studies suggest that microstructural abnormalities also occur [17, 18]. The pathophysiological mechanisms responsible for these changes are unclear. For example, whether all the damage is due to direct effects of smoking, or specific effects of pulmonary and cardiovascular disease, or mechanisms common to both. Furthermore, smoking may interact with other risk factors associated with accelerated age-related brain changes and cognitive decline, including lifestyle, psychosocial factors and co-morbid disease. This analysis was designed to test whether there are common factors contributing to brain changes in COPD and CAD.

## Materials and methods

### Participants

This prospectively planned cross-sectional analysis combined data from two complementary studies that used identical assessment methods to permit combination. 736 patients were assessed for eligibility and 103 enrolled into one of the two studies. Three patients did not have MRI, so were excluded from this analysis. This cohort comprised 53 patients with a primary diagnosis of COPD who had experienced at least one exacerbation in the preceding year and 47 patients with a primary diagnosis of CAD, 23 of whom also had COPD. They were recruited from outpatient respiratory and cardiology departments at St George’s Hospital NHS Foundation Trust between December 2015 and October 2017. They were included if they were aged over 40 years and had at least 10 pack years smoking history. COPD was defined using GOLD criteria. CAD patients were included if they had a Gensini score for coronary atherosclerosis greater than zero (i.e. indicative of coronary artery narrowing) [19]. Exclusion criteria included a primary respiratory diagnosis other than COPD, an exacerbation within the previous six weeks, obstructive sleep apnoea, need for home ventilation, cerebrovascular, neurological or uncontrolled major psychiatric disorder, current alcohol or drug abuse, uncontrolled hypertension, vascular complications of diabetes, hepatic failure, end-stage renal disease, non-cured tumours with prognosis of less than one year, or contraindications for MRI.

Ethical approval was granted by the national research ethics committee London–Dulwich (16/LO/0547) and East Midlands–Leicester South Research Ethics Committee (15/EM/0425). All patients provided informed written consent to participate in this study.

### Clinical and imaging markers

Standard demographic information (age and sex) was collected together with medical history, smoking status and measures representing:

#### Respiratory function

Post-bronchodilator spirometry (Forced Expiratory Volume in 1 second [FEV_1_] and Forced Vital Capacity [FVC]) performed using ATS/ERS criteria, arterial oxygen saturation and MRC dyspnoea score.

#### Vascular risk

Pack year smoking history, diastolic and systolic blood pressure, total cholesterol, high-density lipoprotein (HDL), low-density lipoprotein (LDL), non-HDL cholesterol (i.e., total cholesterol–HDL), body mass index (BMI), glycated haemoglobin (HbA1C), Homeostatic Model Assessment of Insulin Resistance (HOMA-IR).

#### Cardiovascular system markers

Aortic pulse wave velocity assessed using the Vicorder® Arterial Stiffness Model (SMT medical GmbH & Co., Wuerzburg, Germany), serum troponin T and cerebral perfusion (median and peak height of grey matter cerebral blood flow [CBF]) quantified from brain MRI.

#### Systemic inflammation

High-sensitivity C-Reactive Protein (hs-CRP), fibrinogen and total neutrophil count.

#### Microvascular pathology

Quantitative markers of retinal microvascular morphometry including arteriole calibre, venule calibre, arteriole branching angle, venule branching angle, arteriole tortuosity, venule tortuosity and total fractal dimension, as well as urine albumin to creatinine ratio (ACR), a marker of renal microvascular disease.

#### Brain MRI measures

Markers of brain macrostructure (white matter, grey matter, cerebrospinal fluid [CSF], and lateral ventricle volumes), white matter hyperintense lesions (WMHs), and markers of white matter microstructure (median and peak height of white matter fractional anisotropy [FA] and mean diffusivity [MD]).

All measures were acquired during a single visit. Full details of data acquisition and analysis methods for retinal fundus photography and MRI scans are given in S1 Appendix.

### Statistical analysis

#### Preliminary data analysis

To control for possible confounding effects of age and sex all analyses were performed on the covariate adjusted estimates after correcting for these variables. Several variables had a non-Gaussian distribution and were transformed to approximate Guassianity using log_10_ and square root transformations with or without first reflecting about the mean. Pairwise deletion was used for cases with missing data.

Principal Component Analysis (PCA) was used to aggregate appropriate continuous markers into composite measures. Direct oblimin rotation was performed using SPSS (IBM SPSS Statistics 24). Six composite measures were derived from the markers for vascular risk, respiratory function, cardiovascular system, retinal microvascular pathology and systemic inflammation. A similar approach was used to combine the measures of brain structure. Individual variables with less than 0.5 Kaiser-Meyer-Olkin sampling adequacy were removed from the model and the PCA re-calculated. Scree tests (above the ‘elbow’ in the eigenvalue scree plot) were used to determine the number of components to extract. Component scores were computed using least squares regression which took into account each marker’s contribution to the component, the correlations between markers and correlations between components [20]. For markers that did not load onto a component, the measured values (after age and sex adjustment) were used. For clarity, the following naming conventions are used: composite measure names given in Title Case; single marker names given in *italics*.

#### Relationships between disease markers

Pearson’s correlations were performed to investigate univariate relationships between brain structure and markers from other organs and these were summarised in a network diagram generated using Cytoscape (version 3.7.2 https://cytoscape.org/).

A three-step hierarchical regression was then used to test the correlation of each disease marker with each brain measure. Step 1 was a simple correlation with data from both diagnostic groups combined. If there was a significant correlation (*p*<0.05), Step 2 was a test of the effect of including diagnostic group membership. Step 3 tested whether the slope of the relationship was significantly different between the two diseases by inclusion of a diagnostic group-by-disease marker interaction term. Markers that showed a significant correlation with changes in the brain in these univariate analyses, but no difference in slope between the two diseases, were then entered into multivariable linear models.

## Results

### Demographics

Cohort demographics are given in Table 1. The mean age was 68 years, 22% were current smokers. Of those with COPD, the median FEV_1_% predicted was 74 (interquartile range [IQR] 54–92). Hypertension was present in 83%, 5% had type-2 diabetes and 77% had dyslipidaemia. The median Gensini score in patients with CAD was 22 (IQR 13–46). All participants reported some degree of breathlessness (MRC dyspnoea ≥1).

**Table 1:** Demographics and clinical characteristics.

Demographics	CAD (n = 47)	COPD (n = 53)	Whole cohort (n = 100)
Age (years)	68 ± 7	69 ± 8	68 ± 8
Males (%)	77	62	69
Current smokers (%)	13	30	22
**Respiratory function and symptoms**			
FEV_1_% predicted	89 [73–96]	55 [32–77]	74 [54–92]
FVC % predicted	97 ± 19	82 ± 24	89 ± 23
FEV_1_/FVC	0.73 [0.67–0.77]	0.56 [0.39–0.62]	0.65 [0.51–0.73]
Oxygen saturations (%)	97 [96–97]	96 [94–97]	96 [95–97]
MRC dyspnoea	2 [1–2]	3 [2–4]	2 [2–4]
**Vascular risk factors**			
Pack years smoking history	30 [19–45]	45 [27–56]	40 [20–50]
Systolic blood pressure (mm Hg)	140 ± 17	150 ± 16	145 ± 17
Diastolic blood pressure (mm Hg)	73 ± 7	75 ± 10	74 ± 9
Total cholesterol (mmol/L)	5.1 ± 1.2	4.9 ± 1.1	5.0 ± 1.3
HDL (mmol/L)	1.3 [1.0–1.5]	1.6 [1.3–2.3]	1.4 [1.2–1.8]
LDL (mmol/L)	3.0 ± 1.1	2.5 ± 1.1	2.8 ± 1.1
Non-HDL (mmol/L)	3.8 ± 1.1	3.1 ± 1.2	3.4 ± 1.2
Body mass index (kg/m^2^)	28 [26–31]	27 [24–31]	28.0 [24.2–31.0]
HbA1c (mmol/mol)	38 [36–42]	37 [35–41]	38 [35–41]
HOMA-IR	1.23 [0.78–1.84]	1.19 [0.76–1.88]	1.21 [0.79–1.87]
**Cardiovascular function**			
Aortic pulse wave velocity (m/s)	9.8 [8.7–11.3]	9.9 [9.0–11.2]	9.9 [8.8–11.3]
Troponin T (ng/L)	8.5 [6.8–13.0]	2.8 [2.2–4.1]	4.2 [2.7–8.0]
Median CBF (mL/100g/min)	37.2 ± 8.2	41.5 ± 7.4	39.5 ± 0.8
CBF peak height	0.045 ± 0.001	0.041 ± 0.076	0.043 ± 0.009
**Systemic inflammation**			
Hs-CRP (mg/L)	2.4 [1.0–5.7]	3.4 [1.6–6.3]	3.3 [1.2–6.0]
Fibrinogen (g/L)	3.3 [2.9–3.7]	3.7 [3.0–4.4]	3.5 [3.0–4.0]
Neutrophil count x10^9^/L	4.5 [3.5–5.7]	3.3 [3.5–5.7]	4.5 [3.5–5.7]
**Microvasculature**			
Arteriole calibre (CRAE zone C, μm)	147.2 ± 13.1	138.8 ± 14.7	146.9 ± 13.0
Venule calibre (CRVE zone C, μm)	213.5 ± 23.1	208.4 ± 25.9	210.8 ± 22.6
Arteriole tortuosity	1.10 [1.09–1.12]	1.10 [1.09–1.12]	1.10 [1.09–1.12]
Venule tortuosity	1.10 [1.09–1.12]	1.10 [1.09–1.12]	1.10 [1.09–1.12]
Arteriole branching angle (°)	73.5 [64.7–82.6]	77.9 [67.1–82.7]	75.2 [66.8–83.0]
Venule branching angle (°)	74.1 ± 10.2	77.0 ± 11.5	75.4 ± 10.8
Total fractal dimension	0.03 [0.02–0.05]	0.03 [0.02–0.05]	0.03 [0.02–0.05]
Urine ACR	2.2 [0.8–3.6]	1.3 [0.0–3.1]	1.5 [0.4–3.2]
**Brain structure**			
Grey matter volume (% TIV)	40.0 ± 3.0	40.1 ± 3.5	40.0 ± 3.3
White matter volume (% TIV)	28.1 ± 2.7	27.9 ± 2.6	28.0 ± 2.7
CSF volume (% TIV)	31.9 ± 5.0	32.0 ± 5.2	32.0 ± 5.1
Lateral ventricle volume (% TIV)	3.09 [2.41–4.13]	3.24 [2.55–4.31]	3.21 [2.53–4.28]
WMH volume (% TIV)	0.27 [0.11–0.78]	0.36 [0.17–1.26]	0.32 [0.14–0.82]
Median FA	0.41 ± 0.02	0.39 ± 0.03	0.40 ± 0.03
FA peak height	0.026 ± 0.002	0.029 ± 0.002	0.028 ± 0.002
Median MD (×10^-3^mm^2^ s^-1^)	0.72 ± 0.03	0.75 ± 0.03	0.74 ± 0.03
MD peak height	0.14 ± 0.02	1.14 ± 0.02	0.14 ± 0.02

Demographic and clinical characteristics. For data with a Gaussian distribution across the whole cohort, means ± standard deviations are presented, for frequency data percentages are presented and for non-Gaussian data medians and upper and lower quartiles [Q1-Q3] are presented. Median and normalised peak heights for the distribution of MD and FA values within the normal appearing white matter are reported. ACR = Albumin to Creatinine Ratio, CBF = Cerebral Blood Flow, COPD = Chronic Obstructive Pulmonary Disease, CRAE = Central Retinal Artery Equivalent, CRVE = Central Retinal Vein Equivalent, CSF = Cerebrospinal Fluid, FA = Fractional Anisotropy, FEV_*1*_ = Forced Expiratory Volume in 1 second, FVC = Forced Vital Capacity, HbA1c = Glycated Haemoglobin, HDL = High-Density Lipoprotein, HOMA-IR = Homeostatic Model Assessment of Insulin Resistance, Hs-CRP = high-sensitivity C-Reactive Protein, LDL = Low-Density Lipoprotein, MD = Mean Diffusivity, MRC = Medical Research Council, TIV = Total Intracranial Volume, WMH = White matter hyperintense lesion.

### Principal component analysis

Naming and interpretation of components was based on marker variables with high component loadings (>0.6). Markers loaded positively onto a component unless stated otherwise.

#### Respiratory function

One component was extracted (Respiratory Function) which explained 75.4% of the total variance. It contained FEV_1_% predicted, FVC % predicted and SaO_2_. MRC dyspnoea was retained as a separate variable to provide a measure of respiratory symptoms (see S1 Table in S2 Appendix).

#### Vascular risk

Three components were extracted from the vascular risk factors: Plasma Lipids, comprising Non-HDL (negative loading) and HDL; Blood Pressure comprising systolic blood pressure and diastolic blood pressure; Glucose Dysregulation comprising HbA1C, HOMA-IR and BMI. These explained 71.4% of the total variance of the model (Glucose Dysregulation = 33.8%, Blood Pressure 23.1%, Plasma Lipids 14.6%) (see S2 Table in S2 Appendix). Pack years did not fit any component so was retained as a separate variable.

#### Cardiovascular system

These markers did not form a single composite measure, so were analysed as individual markers.

#### Systemic inflammation

These markers did not form a single composite measure so were analysed as individual markers.

#### Retinal microvascular pathology

Retinal microvascular data was unavailable for 16 patients due to ungradable retinal images. Two components were extracted: Retinal Vessel Calibre, comprising arteriole calibre and venule calibre and Retinal Vessel Tortuosity, comprising arteriole tortuosity, venule tortuosity and total fractal dimension (negative loading). These explained 56.0% of the total variance of the model (Retinal Vessel Calibre 29.5%, Retinal Vessel Tortuosity 26.5%). Both were used for the tests of association with brain structure (see S3 Table in S2 Appendix). Arteriole branching angle did not fit any component and was not considered further.

#### Brain structure

Two principal components were extracted. One was Brain Tissue Volume, composed of grey matter volume (which had negative loading) and CSF and lateral ventricle volumes that had positive loadings. The other was White Matter Microstructure composed of FA peak height, median FA (both with negative loading), and median MD with a positive loading. Together, these components explained 80.5% of the total variance of the model (White Matter Microstructure 48.6%, Brain Tissue Volume 31.9%). An initial PCA found that WMH volume loaded approximately equally onto both brain components, so it was removed from the PCA and analysed as a separate brain variable (see S4 Table in S2 Appendix).

#### Single variables

The following disease markers were analysed as single variables: *MRC dyspnoea*, *pack years smoking*, *troponin T*, *aortic pulse wave velocity*, *median CBF*, *CBF peak height*, *urine ACR*, *hs-CRP*, *fibrinogen* and *neutrophil count*.

### Correlation analysis

#### Association between disease markers outside of the brain

Significant correlations between the different disease markers were infrequent and, where present, were generally weak (|*r|* = 0.21 to 0.42). Several relationships with Plasma Lipids were the reverse of what was expected and probably because lipid lowering treatment was given to patients with high serum cholesterol (see S5 Table in S2 Appendix). Of note, a significant correlation was found between higher urine ACR and lower median CBF (*r* = 0.273, *p* = 0.027).

#### Univariate association between brain structure and disease markers

**Fig 1:**
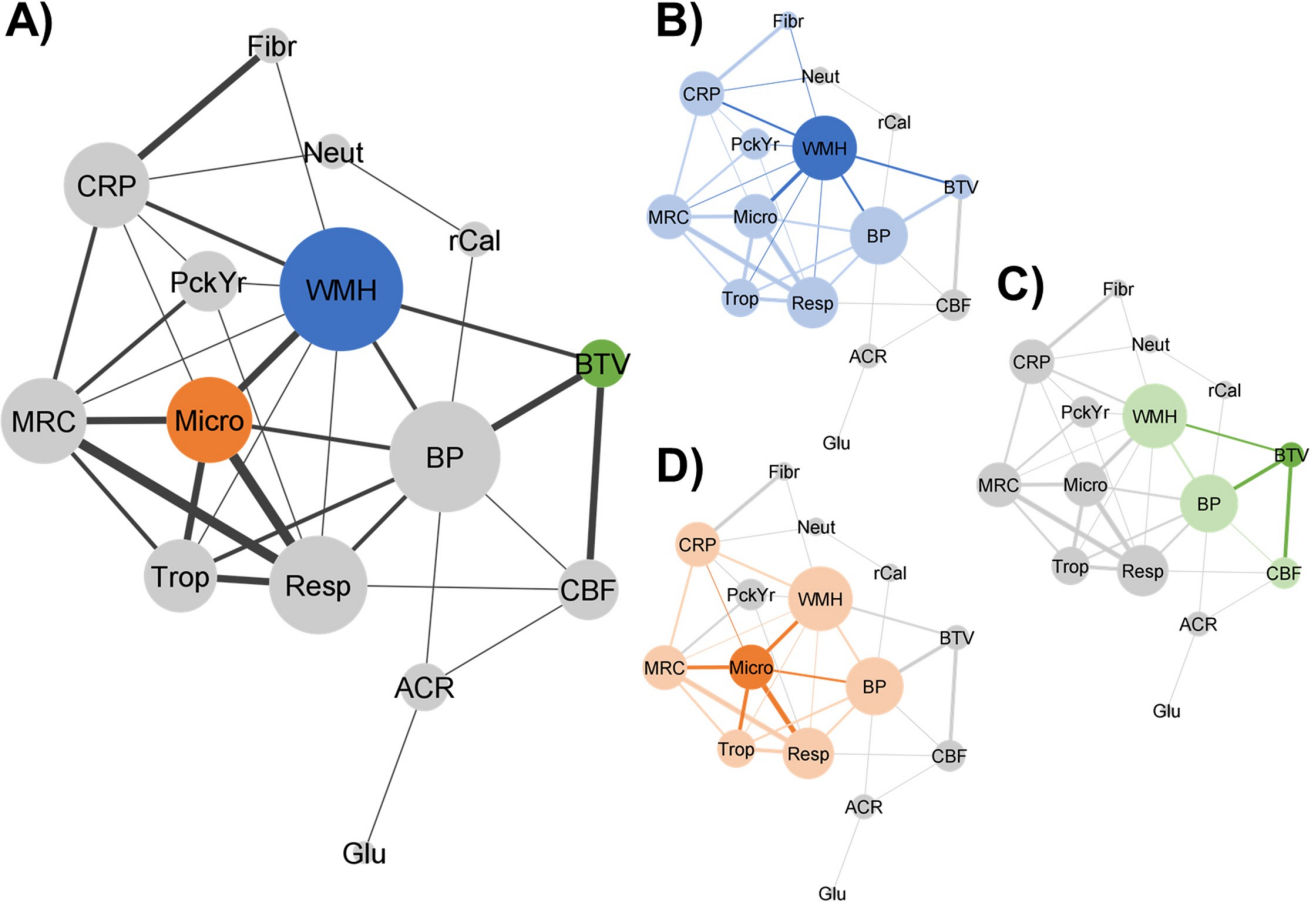
Network diagram. Pearson’s correlations between non-brain and brain measures (circles) are represented as network connections. All lines indicate significance at *p*<0.05, line thickness indicates strength of correlation (correlation coefficient). (A) whole network. (B) Brain Tissue Volume (green), (C) white matter hyperintensity volume (blue), (D) White Matter Microstructure (orange). Dark colours show direct correlations with a brain measure. Light colours indicate intercorrelations between non-brain measures. Circle size reflects the number of connections. Relationships with CBF peak height and Plasma Lipids are not shown. ACR = Urine Albumin to Creatinine Ratio, BTV = Brain Tissue Volume, BP = Blood Pressure, CBF = median cerebral blood flow, CRP = high-sensitivity C-Reactive Protein, Fibr = Fibrinogen, Glu = Glucose Dysregulation, Micro = Lower White Matter Microstructure, MRC = MRC dyspnoea, Neut = Neutrophils, PckYr = pack year smoking history, Resp = Respiratory Function, rCal = Retinal Vessel Calibre, rTor = Retinal Vessel Tortuosity, Trop = troponin T, WMH = white matter hyperintense lesion volume.

A number of correlations were found between markers of brain structure and disease markers in other organs. This is summarised in a network diagram (Fig 1A). The subnetworks show the specific markers that were correlated with Brain Tissue Volume (Fig 1B), WMH Volume (Fig 1C) and White Matter Microstructure (Fig 1D). It will be noted that retinal markers did not correlate with any brain measures.

**The relationship between Brain Tissue Volume** and the disease markers was explored using hierarchical regression (full details in S6 Table in S2 Appendix), the significant relationships are shown in Table 2. This component was significantly associated with *median CBF*, Blood Pressure and *CBF peak height*, such that higher component scores (greater CSF volume and lower grey matter volume) were associated with lower *median CBF*, higher blood pressure and higher *CBF peak height*. In each of the comparisons there was no significant difference between the two diagnostic groups. Similarly, there was no difference in slope of the relationships between diagnostic groups, as shown at Step 3.

**Table 2:** Single variable models (hierarchical linear regression)–Brain Tissue Volume.

Predictor		Main Effect (Disease marker)		Main Effect (Group)		Interaction (Group × Disease marker)		Overall model		
	Step	*t*	*p*	*t*	*p*	*t*	*p*	adj. *r*^2^	*F*	*p*
Blood Pressure^†^	1	4.133	<0.001					0.153	17.082	<0.001
	2	4.399	<0.001	-1.440	0.153			0.163	9.682	<0.001
	3	2.078	0.041	-1.344	0.182	0.534	0.595	0.156	6.497	0.001
Median CBF	1	-4.795	<0.001					0.191	22.989	<0.001
	2	-4.827	<0.001	0.834	0.406			0.189	11.804	<0.001
	3	-3.507	0.001	0.840	0.403	-0.278	0.782	0.180	7.816	<0.001
CBF peak height	1	3.736	<0.001					0.122	13.961	<0.001
	2	3.725	<0.001	0.549	0.584			0.116	7.078	0.001
	3	2.483	0.015	0.545	0.587	0.338	0.736	0.107	4.711	0.004

Three-step hierarchical linear model showing the relationship between Brain Tissue Volume and disease markers. Step 1 predictors: disease marker only; Step 2 predictors: disease marker and diagnostic group (Group); Step 3 predictors: disease marker, diagnostic group, diagnostic group × disease marker interaction. Reported are the *t*-statistics (*t*) and *p*-values for each main effect and interaction, as well as the regression coefficient (*r*^2^), adjusted regression coefficient (adj *r*^2^), *F*-statistics (*F*) and *p*-values (*p*) for the overall models. The COPD>CAD contrast for the main effect of group and the group × disease marker interaction is shown. All models included a constant term (not shown). All variables were adjusted for age and sex. CBF = Cerebral Blood Flow.

^†^Principal component.

Higher **WMH volume** was significantly associated with lower Respiratory Function, and higher *MRC dyspnoea*, *pack years*, Blood Pressure, *troponin T*, *fibrinogen* and *hs-CRP*; full details of all analyses are presented in S7 Table in S2 Appendix, and significant relationships are shown in Table 3. Respiratory Function, *MRC dyspnoea*, Blood Pressure, *troponin T*, and *hs-CRP* showed no significant effect of disease group.

**Table 3:** Single variable models (hierarchical linear regression)–WMH volume.

Predictor		Main Effect (Disease marker)		Main Effect (Group)		Interaction (Group × Disease marker)		Overall model		
	Step	*t*	*P*	*t*	*p*	*t*	*p*	adj. *r*^2^	*F*	*p*
Respiratory Function^†^	1	-2.559	0.012					0.052	6.550	0.012
	2	-2.501	0.014	-0.616	0.539			0.048	3.444	0.036
	3	-1.466	0.146	-0.676	0.501	0.292	0.771	0.039	2.302	0.082
MRC dyspnoea	1	2.430	0.017					0.048	5.906	0.017
	2	2.345	0.021	-0.714	0.477			0.043	3.193	0.045
	3	0.596	0.533	-0.413	0.681	0.588	0.558	0.036	2.230	0.090
Pack years (square root)	1	1.985	0.050					0.029	3.939	0.050
	2	1.787	0.077	0.396	0.693			0.021	2.031	0.137
	3	1.042	0.300	0.398	0.691	0.069	0.945	0.010	1.342	0.266
Blood Pressure^†^	1	3.034	0.003					0.084	9.206	0.003
	2	2.830	0.006	0.137	0.892			0.074	4.561	0.013
	3	2.144	0.035	0.045	0.965	-0.640	0.524	0.068	3.157	0.029
Troponin T (log_10_)	1	2.443	0.017					0.052	5.967	0.017
	2	2.066	0.042	0.522	0.603			0.044	3.096	0.050
	3	2.197	0.031	0.230	0.819	-1.291	0.200	0.052	2.635	0.055
Hs-CRP (log_10_)	1	3.125	0.002					0.084	9.765	0.002
	2	3.082	0.003	0.885	0.378			0.082	5.263	0.007
	3	2.840	0.006	0.888	0.377	-0.980	0.330	0.081	3.827	0.012

Three-step hierarchical linear model showing the relationship between WMH volume and disease markers. Step 1 predictors: disease marker only; Step 2 predictors: disease marker, group; Step 3 predictors: disease marker, diagnostic group (Group), diagnostic group × disease marker interaction. Reported are the *t*-statistics (*t*) and *p*-values for each main effect and interaction, as well as the regression coefficient (*r*^2^), adjusted regression coefficient (adj *r*^2^), *F*-statistics (*F*) and *p*-values (*p*) for the overall models. The COPD>CAD contrast for the main effect of group and the group × disease marker interaction is shown. All models included a constant term (not shown). All variables were adjusted for age and sex. Hs-CRP = high-sensitivity C-Reactive Protein, MRC = Medical Research Council, WMH = White matter hyperintense lesion.

^†^Principal component.

The models for **White Matter Microstructure** showed that less well organised structure was associated with lower Respiratory Function and higher *MRC dyspnoea score*, Blood Pressure, *troponin T* and *hs-CRP*. The full results can be found in S8 Table in S2 Appendix and significant relationships are shown in Table 4. There was a consistent significant effect of group membership at Step 2; COPD patients having on average less well organised white matter than CAD patients. Disease markers that still retained a significant effect of the disease marker after inclusion of the disease group effect were carried through to the multivariable model.

**Table 4:** Single variable models (hierarchical linear regression)–White Matter Microstructure.

Predictor		Main Effect (Disease marker)		Main Effect (Group)		Interaction (Group × Disease marker)		Overall model		
	Step	*t*	*P*	*t*	*p*	*t*	*p*	adj. *r*^2^	*F*	*p*
Respiratory Function^†^	1	-6.335	<0.001					0.287	40.126	<0.001
	2	-4.133	<0.001	2.855	0.005			0.337	25.634	<0.001
	3	-1.797	0.076	2.757	0.007	-0.234	0.816	0.330	16.938	<0.001
MRC dyspnoea	1	5.084	<0.001					0.202	25.847	<0.001
	2	2.290	0.024	2.999	0.003			0.263	18.484	<0.001
	3	2.138	0.035	2.266	0.026	-1.188	0.238	0.266	12.846	<0.001
Blood Pressure^†^	1	3.984	<0.001					0.143	15.875	<0.001
	2	2.895	0.005	3.883	<0.001			0.261	16.745	<0.001
	3	1.180	0.241	3.911	<0.001	0.582	0.562	0.256	11.191	<0.001
Troponin T (log_10_)	1	4.796	<0.001					0.196	23.01	<0.001
	2	3.353	0.001	3.886	<0.001			0.306	20.876	<0.001
	3	1.743	0.085	3.779	<0.001	0.039	0.969	0.298	13.760	<0.001
Hs-CRP (log_10_)	1	2.142	0.035					0.036	4.587	0.035
	2	2.221	0.029	5.552	<0.001			0.266	18.428	<0.001
	3	2.551	0.012	5.585	<0.001	-1.394	0.167	0.274	13.056	<0.001

Three-step hierarchical linear model showing the relationship between White Matter Microstructure and disease markers. Step 1 predictors: disease marker only; Step 2 predictors: disease marker, group (Group); Step 3 predictors: disease marker, group, group × disease marker interaction. Reported are the *t*-statistics (*t*) and *p*-values for each main effect and interaction, as well as the regression coefficient (*r*^2^), adjusted regression coefficient (adj *r*^2^), *F*-statistics (*F*) and *p*-values (*p*) for the overall models. The COPD>CAD contrast for the main effect of group and the group × disease marker interaction is shown. All models included a constant term (not shown). All variables were adjusted for age and sex. Hs-CRP = high-sensitivity C-Reactive Protein, MRC = Medical Research Council.

^†^Principal component.

### Multi-variable associations between brain structure and disease markers

**Fig 2:**
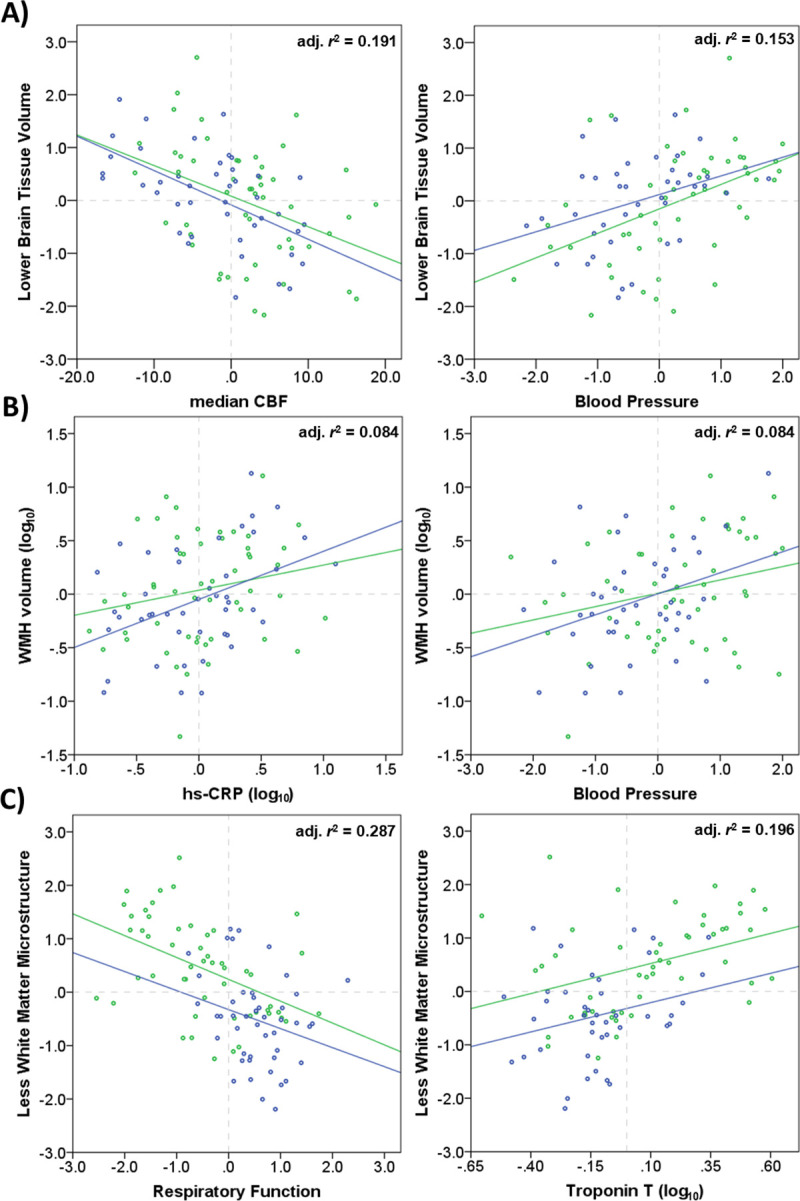
Relationships between brain structure and other disease markers. Plots showing the relationship between: Brain Tissue Volume, median CBF and Blood Pressure (panel A); WMH volume, hs-CRP and Blood Pressure (panel B); and White Matter Microstructure, Respiratory Function and troponin T (panel C) for COPD (green) and CAD (blue) patients. The adjusted r^2^ goodness of fit is presented for the whole cohort. Data has been adjusted for age and sex. CBF = cerebral blood flow, hs-CRP = high-sensitivity C-Reactive Protein, WMH = white matter hyperintense lesion.

The effect of *Median CBF* and *CBF peak height* on Brain Tissue Volume was found to be collinear, so only Blood Pressure and *median CBF*, were included as predictors in the multivariable model. Both were independent predictors: *median CBF* (t = -3.592, *p*<0.001) and Blood Pressure (*t* = 3.356, *p =* 0.001), Table 5. The model explained 30% of the total variance in Brain Tissue Volume, adjusted *r*^2^ = 0.279, *p*<0.001. Scatterplots showing the univariate relationship between Brain Tissue Volume and these markers are shown in Fig 2A.

**Table 5:** Multivariable models of brain structure.

**Brain Tissue Volume**					
Predictor	*B* [95% Confidence Intervals]		*SE*	*t*	*p*
Constant	0.005 [-0.178, 0.188]		0.092	0.057	0.955
Blood Pressure^†^	0.311 [0.127, 0.496]		0.093	3.356	0.001
Median CBF	-0.048 [-0.073, -0.024]		0.012	-3.592	<0.001
	*r* ^2^	adj. *r*^2^	SE	*F*	*p*
Overall model	0.297	0.279	0.848	17.286	<0.001
**WMH Volume**					
Predictor	*B* [95% Confidence Intervals]		*SE*	*t*	*p*
Constant	-0.001 [-0.099, 0.097]		0.049	-0.030	0.977
Respiratory Function^†^	-0.032 [-0.166, 0.102]		0.067	-0.473	0.637
MRC dyspnoea	0.019 [-0.084, 0.122]		0.052	0.366	0.715
Blood Pressure^†^	0.114 [0.007, 0.223]		0.054	2.129	0.036
Troponin T (log_10_)	0.187 [-0.209, 0.608]		0.205	0.971	0.335
Hs-CRP (log_10_)	0.247 [0.074, 0.546]		0.118	2.619	0.011
	*r* ^2^	adj. *r*^2^	*SE*	*F*	*p*
Overall model	0.216	0.165	0.448	4.246	0.002
**White Matter Microstructure**					
Predictor	*B* [95% Confidence Intervals]		*SE*	*t*	*p*
Constant	-0.213 [-0.513, 0.087]		0.151	-1.415	0.199
Group	0.397[-0.072, 0.865]		0.235	1.687	0.096
Respiratory Function^†^	-0.262 [-0.495, -0.028]		0.117	-2.234	0.028
MRC dyspnoea	0.029 [-0.175, -0.233]		0.103	0.282	0.779
Blood Pressure^†^	0.164 [-0.027, 0.356]		0.096	1.710	0.091
Troponin T (log_10_)	0.694 [-0.012, 1.401]		0.355	1.958	0.054
Hs-CRP (log_10_)	0.366 [-0.047, 0.778]		0.207	1.765	0.082
	*r* ^2^	adj. *r*^2^	*SE*	*F*	*P*
Overall model	0.445	0.402	0.773	10.177	<0.001

Multiple linear regression results showing relationships between disease markers and measures of brain structure. Reported are the unstandardised beta coefficients *(B)*, their 95% confidence intervals and standard error estimates (SE), the *t*-statistics (*t*) and *p*-values for each main effect and the regression coefficient (*r*^2^), adjusted regression coefficient (adj *r*^2^), SE, *F*-statistics (*F*) and *p*-values (*p*) for each overall model. All variables were adjusted for age and sex. CBF = Cerebral Blood Flow, Hs-CRP = high-sensitivity C-Reactive Protein, MRC = Medical Research Council, WMH = White matter hyperintense lesion.

^†^Principal component.

The multivariable model of *WMH volume* contained Respiratory Function, *MRC dyspnoea*, Blood Pressure, *troponin T*, and *hs-CRP* explained 22% of the total variance (adjusted *r*^2^ = 0.165, *p* = 0.002). Blood Pressure (*t* = 2.129. *p* = 0.036) and *hs-CRP* (*t* = 2.619. *p* = 0.011) remained as significant independent predictors (Table 5). Scatterplots showing the relationship between WMH volume and Blood Pressure and *hs-CRP* are shown in Fig 2B.

The multivariable model for White Matter Microstructure contained Respiratory Function, *MRC dyspnoea score*, Blood Pressure, *troponin T* and *hs-CRP* and explained 45% of the variance (adjusted *r*^2^ = 0.402, *p*<0.001). Only Respiratory Function (*t* = -2.234, *p* = 0.028) remained as significant independent predictor of White Matter Microstructure (Table 5), although *troponin T* approached significance (*p =* 0.054). Scatterplots showing the relationship between White Matter Microstructure and Respiratory Function, *troponin T* and are shown in Fig 2C.

## Discussion

This study was designed to test whether there were common factors linking COPD and CAD to brain changes. A large number of correlations were found between brain MRI markers and markers of disease in the respiratory, cardiovascular and inflammatory systems and the slope of these relationships was not different between COPD and CAD. This suggests that there are common mechanisms linking these two diseases to changes in the brain. Multivariable models showed that lower brain tissue volumes were significantly associated with higher blood pressure and lower CBF as independent predictors. Greater WMH volume was associated with higher blood pressure and higher hs-CRP. Less well organised white matter microstructure was significantly associated with lower respiratory function and, possibly, serum troponin T. It is noteworthy that the amount of previous smoking was not an independent predictor of any brain marker when other factors were considered. It is not possible to evaluate the degree to which smoking directly causes brain damage rather than through indirect effects on other organs, however, no evidence was found of an independent effect of amount of smoking beyond those related to markers of respiratory and cardiovascular disease. Although causality cannot be determined in this cross-sectional study, the findings suggest the presence of pulmonary, inflammatory and vascular aetiologies for brain changes in these patients with smoking-related heart and lung disease.

The pathway between lung and brain changes is not clear, but our findings suggest that the link is not simply due to a shared smoking aetiology. This study along with others [3, 21], suggests that structural brain changes can occur in COPD independently of cardiovascular risk. Hypoxaemia may be a factor, but cognitive impairment has been reported in non-hypoxemic COPD patients [22] and in our study only seven individuals had mild hypoxia. Episodic nocturnal, exertional or exacerbation-related desaturation may have an effect since similar cognitive changes and brain changes have also been reported in sleep apnoea [23, 24], however cardiovascular disease is also a common comorbidity in that condition and may be an important factor.

We found univariate correlations between higher CRP and fibrinogen and WMH volume and higher CRP and less well organised white matter microstructure, but only the relationship between CRP and WMH volume remained significant in the multivariable models. CRP is an acute-phase protein synthesised in response to pro-inflammatory cytokines during infection and injury. It has been suggested that inflammation provides the link between smoking and the development of systemic disease [25]. Tobacco smoking promotes a broad range of pulmonary and systemic immune changes [26] and COPD is associated with enhanced inflammatory responses in the lungs, systemic inflammation and low-level vascular inflammation [27]. Once established, these inflammatory processes persist beyond smoking cessation [25, 28]. Chronic systemic inflammation has been associated with greater burden of atherosclerotic disease and elevated risk of ischaemic stroke within the general population (see review [29]). Other studies have also reported associations between systemic inflammation, incident brain infarcts, WMH severity, white matter microstructural abnormalities [30–32] and microcirculatory dysfunction [33]. Therefore, it is thought that systemic inflammation plays an aetiological role in cerebral small vessel disease [30], via endothelial dysfunction, subsequent impairment of blood flow and increased blood-brain permeability [34]. Our patients were studied in the stable state, but systemic inflammation may be episodic as COPD exacerbations are associated with increases in fibrinogen [35] and platelet aggregation [36].

The finding of relationships between blood pressure and MRI markers of brain structure supports previously reported associations between hypertension and WMHs [37], cerebral microbleeds [38], brain infarcts [39], white matter microstructural damage [40] and cerebral atrophy [41]. Prolonged hypertension may lead to accelerated age-related changes in brain structure and function by promoting small vessel wall remodelling and endothelial dysfunction leading to compromised autoregulation and leaving the brain vulnerable to hypoperfusion or hypoxaemia; however, one study reported that cardiovascular risk factors only explained approximately 2% of WMH variance in older adults [42].

An association was found between lower brain tissue volume and lower CBF, which is consistent with the adverse effects of chronically reduced CBF in neurodegenerative disorders related to ageing and cardiovascular disease [43]. Reductions in CBF have also been found to occur in chronic smokers [44], with proposed mechanisms that include smoking-induced oxidative stress, endothelial dysfunction, nitric oxide vasodilatation, neurovascular coupling, reduction in cardiac output, hypocapnia, and autoregulatory dysfunction [44].

We found no relationships between retinal and kidney microvascular markers and brain MRI markers. This was unexpected, given that previous studies reported retinal microvascular changes in chronic smokers and COPD patients [45, 46] and moderate associations between retinal markers and features of cerebral small vessel disease (infarcts, cerebral microbleeds and WMHs), cognitive impairment and dementia (see [47], for a review). It is also possible that this study was underpowered to detect these effects, since there was missing data for retinal variables. However, we did find a correlation between higher urine ACR and lower CBF (*r* = 0.273, *p* = 0.027, see online supplement), therefore, it is possible that cerebral hypoperfusion may have mediated at least part of the previously reported relationship between markers of microvascular disease and changes in brain structure.

### Strengths and limitations

This study benefited from a comprehensive range of clinical measures encompassing multiple end-organs, and high-resolution multi-modal brain MRI. This allowed investigation of a range of possible relationships between disease markers from different organ systems and the brain. Despite this, a large amount of variance in the different types of brain marker remained unaccounted for. A number of factors that might contribute to neurodegeneration in smoking and COPD were not tested. These include hypercapnia, nocturnal and exertional desaturation, oxidative stress and direct neurotoxic effects from tobacco smoke, including carbon monoxide.

The absence of a healthy control group means that it is not possible to determine whether the relationships found in this study are specific to smoking-related diseases or are more widely applicable to the general population. However, the pattern of changes is similar to those previously reported in the brains of COPD patients compared to healthy controls [3, 21]. Furthermore, white matter hyperintensity lesions are indicative of white matter damage [48]. In this study, we showed a correlation between the volume of these lesions and well-established markers of disease such as lung function, breathlessness, blood pressure, troponin and CRP. We also carried out all analyses after adjusting for age, so it is reasonable to conclude that the associations that we found are a feature of disease and not an aspect of aging in otherwise healthy people. The statistical analyses and generalisability of this study were limited by the relatively small sample size (though large compared to similar brain imaging studies in smoking-related lung disease). The study was designed to test a specific primary hypothesis—that there are common mediators between COPD and CAD that produce changes in brain structure. That required a number of tests without correction for multiple comparisons, which increased the risk of Type-I error and some of the relationships may be spurious, but we used a hierarchical statistical approach to reduce this risk as far as possible. Conversely, there was also considerable shared variance between the risk factors identified in this study, which could have led to over-adjustment in the multiple linear regression analyses and reduced the estimated degree of association. Structural equation modelling may reduce this bias and provide insight into causal relationships, but it requires large datasets.

The cross-sectional nature of the comparisons limits the inferences that can be drawn about causality, particularly the possible influence of treatment. This is exemplified by the case of lipid lowering drugs such as statins. Several relationships with plasma lipids were the reverse of what might be expected, probably because lipid lowering treatment was given to patients with high serum cholesterol. Thus, any analysis of the relationship between treatment and brain changes could be confounded by treatment indication and treatment duration.

## Conclusions

In both COPD and CAD patients, impaired higher blood pressure and raised serum CRP appear to be independent risk factors for white matter lesion volume. Associations were also found between lower respiratory function and less organised white matter microstructure and between higher blood pressure, lower cerebral blood flow and lower brain tissue volumes. A large amount of variance in brain structure remained unaccounted, suggesting that other factors may also contribute. These findings suggest that multiple preventative or therapeutic interventions may be required to target a range of pathophysiological mechanisms in any attempts to reduce neurodegeneration and cognitive impairment in people with smoking-related diseases.

## Supporting information

S1 Appendix Supplementary methods. Details of data acquisition and analysis methods for retinal fundus photography and MRI scans.
(DOCX)

S2 Appendix Supplementary results: S1-S8 Tables. (DOCX)

## List of abbreviations

ACR: albumin to creatinine ratio
ATS/ERS: American Thoracic Society/European Respiratory Society
BMI: Body Mass Index
CAD: Coronary Artery Disease
CBF: Cerebral Blood Flow
CI: Confidence Interval
COPD: Chronic Obstructive Pulmonary Disease
CRAE: Central Retinal Artery Equivalent
CRP: C-Reactive Protein
CRVE: Central Retinal Vein Equivalent
CSF: Cerebrospinal Fluid
DTI: Diffusion Tensor Imaging
FA: Fractional Anisotropy
FEV_1_: Forced Expiratory Volume in 1 Second
FVC: Forced Vital Capacity
GOLD: The Global Initiative for Chronic Obstructive Lung Disease
HbA1C: Glycated Haemoglobin
HDL: High-Density Lipoprotein
IQR: Interquartile Range
HOMA-IR: Homeostatic Model Assessment of Insulin Resistance
hs-CRP: High-Sensitivity C-Reactive Protein
LDL: Low-Density Lipoprotein
MD: Mean Diffusivity
MRC: Medical Research Council
MRI: Magnetic Resonance Imaging
PCA: Principal Component Analysis
SaO_2_: Oxygen Saturation
TIV: Total Intracranial Volume
WMH(s): White Matter Hyperintensities
