# Supplementary material for: Factors affecting brain structure in smoking-related diseases: Chronic Obstructive Pulmonary Disease (COPD) and coronary artery disease
Source: PLoS One. 2021 Nov 5;16(11):e0259375. doi: 10.1371/journal.pone.0259375 (PMC8570465; doi:10.1371/journal.pone.0259375)
Supplement: S1 Appendix — Details of data acquisition and analysis methods for retinal fundus photography and MRI scans. (DOCX) [file pone.0259375.s001.docx]

# **Supplementary methods**

## **Retinal fundus photography and quantitative measurements of the retinal microvascular morphometry**

Fundus photography was performed using a digital retinal camera system (Topcon 50IX, Topcon Medical systems Inc, Paramus, NJ, USA) after pupil dilation. A retinal image was obtained for each eye (50 degrees of the macula and 35 degrees of the optic disc). A single image was analysed for each person, preferentially using the right eye, unless images were not gradable, in which case images from the left eye were used. A semi-automated computer assisted program (Singapore 1 Vessel Assessment, SIVA [1]) was used to quantitatively assess vessel calibre (central retinal artery equivalent, CRAE and central retinal venule equivalent, CRVE, for zone C), vascular tortuosity (arteriole tortuosity and venule tortuosity), vascular branching angle (arteriole branching angle and venule branching angle) and total fractal dimension [1,2].

## **MRI acquisition**

Whole-brain T1-weighted, fluid-attenuated inversion recovery (FLAIR), diffusion tensor imaging (DTI) and pseudo-continuous arterial spin labelling (pCASL) scans were obtained using a 3-Tesla Phillips Achieva dual TX Magnetic Resonance system.

High resolution sagittal 3D T1-weighted images were acquired using a fast field echo sequence with an echo time (TE) = 3.7ms, repetition time (TR) = 7.87ms, flip angle = 8°, slice thickness = 1.25mm, voxel resolution = 1mm × 1mm × 1.25mm, field-of-view = 240mm × 240mm × 160mm (acquired in 6 minutes, 2 seconds). Axial FLAIR images were acquired using a fast spin echo sequence with spectral pre-saturation with inversion recovery fat suppression, TE = 125ms, TR = 11000ms, inversion time = 2800ms, flip angle = 90°, across 60 slices, slice thickness = 3mm, field-of-view = 230mm × 230mm × 180mm with an in-plane voxel resolution of 1mm × 1mm, giving a voxel resolution of 1mm × 1mm × 3mm (acquired in 5 minutes, 8 seconds).

Axial DTI were acquired using single shot spin echo planar imaging with TE = 75ms, TR = 6450ms, flip angle = 90^o^, voxel resolution = 2mm^3^, field-of-view = 224mm × 224mm × 110mm and acquisition matrix = 112 × 112, providing isotropic 2mm voxel resolution. Eight volumes were acquired without diffusion sensitisation (b = 0 s mm^-2^) and diffusion-weighted images were acquired in 32 non-collinear directions (b = 1000 s mm^-2^). Data were acquired twice to improve signal to noise ratios. DTI were acquired in 9 minutes 49 seconds.

Axial pCASL data and an accompanying proton density weighting image were acquired with single shot echo planar imaging, providing images with slice thickness = 6mm, slice gap = 1mm, field-of-view = 256mm × 256mm × 111mm and voxel resolution 4mm × 4mm × 6mm with a 1mm slice gap. The pCASL were acquired with TE = 8.9ms, TR = 4300ms, flip angle = 90^o^, labelling duration = 1800ms, post-labelling delay = 2000ms, distance between labelling and imaging plane = 85mm and comprised 60 tag-control pairs (acquired in 8 minutes, 45 seconds). Proton density weighted images were acquired with the same image resolution as pCASL with TE = 9ms, TR = 5000ms and flip angle = 90^o^ in 40 seconds.

## **MRI processing**

### **Brain macrostructure**

T1-weighted images were segmented into supra-tentorial grey matter, white matter and cerebrospinal fluid (CSF) tissue probability maps using a semi-automatic procedure adapted from the standard SPM segmentation and normalisation procedure (see [3,4]) (SPM version 12, <https://www.fil.ion.ucl.ac.uk/spm/>). Part of this procedure requires repairing the tissue probability maps for voxels misclassified as grey matter due to the presence of white matter hyperintensities (WMHs). In order to perform this step WMHs were delineated on FLAIR using the semi-automatic contour function in Jim (Jim version 7, Xinapse Systems, <http://www.xinapse.com>) using the definition described in Dodd et al. [5]. Binary WMH masks were produced (i.e. WMH = 1, non-WMH = 0). WMH volume was quantified using Jim (Jim version 7, Xinapse Systems, <http://www.xinapse.com>). FLAIR images were co-registered with the T1-weighted images using affine transformations calculated with ANTS [6] (ANTS, <http://stnava.github.io/ANTs/>). These transformations were applied to the binary WMH masks aligning them with the T1-weighted images. Grey matter, white matter and CSF tissue probability maps were repaired for WMHs - voxels located within the WMH masks were assigned a white matter tissue probability of one and grey matter and CSF, tissue probabilities of zero. Grey matter, white matter and CSF volumes were quantified by integrating tissue probabilities greater than 0.1 within the respective tissue probability maps. Additionally, the bilateral lateral ventricles were automatically parcellated using SIENAX and their volume calculated [7] (FSL version, 5.010, <https://fsl.fmrib.ox.ac.uk/fsl/>). Grey matter, white matter, CSF, WMH and lateral ventricle volumes were normalised for head size by quantifying them as a percentage of total intracranial volume (grey matter + white matter + CSF volumes).

### **White matter microstructure**

DTI were corrected for movement and eddy-current distortions using FSL’s ‘eddy_correct’ [7] (FSL version, 5.010, <https://fsl.fmrib.ox.ac.uk/fsl/>). Fractional anisotropy (FA) and mean diffusivity (MD) were quantified by fitting the diffusion tensor model at every voxel within the DTI using ‘DTIFit’ (FSL version, 5.010, <https://fsl.fmrib.ox.ac.uk/fsl/>). DTI were co-registered with the T1-weighted images using boundary-based registration [8] (FSL version, 5.010, <https://fsl.fmrib.ox.ac.uk/fsl/>). The inverse of these transformations was applied to the repaired tissue probability maps aligning them with the DTI. FLAIR were co-registered with the DTI using FLIRT [9]. These transformations were applied to the WMH masks aligning them with the DTI. WMH masks were re-binarised at voxel values > 0.25. Normal-appearing white matter was defined on the DTI as voxels with a maximum tissue likelihood of being white matter after excluding voxels classified as WMHs. For each patient, the median and normalised peak height of the empirical distribution of FA and MD values within the normal-appearing white matter, were calculated. The normalised peak height is defined as the probability of the mode of the histogram.

### **Cerebral blood flow**

pCASL images were motion corrected via intra-series co-registration to the middle volume of the series using ‘eddy_correct’ [9]) (FSL version, 5.010, <https://fsl.fmrib.ox.ac.uk/fsl/>). Proton density weighted images were aligned to the average of the motion-corrected pCASL time series using FLIRT [9] (FSL version, 5.010, <https://fsl.fmrib.ox.ac.uk/fsl/>). Voxel-wise cerebral blood flow (CBF) was calculated in absolute physiological units from the pCASL images using FSL’s Oxford_ASL [10] (FSL version, 5.010, <https://fsl.fmrib.ox.ac.uk/fsl/>). The ASL kinetic model [11] was applied to each image voxel. The mean difference in magnetisation was averaged over all tag-control pairs and divided by the proton density weighted intensity. To enable CBF quantification several parameters were set according to the consensus recommendations of the ISMRM Perfusion Study Group and the European Consortium for ASL in Dementia [12]. The blood-brain partition coefficient was assumed to be 0.9 ml/g, the labelling efficiency, 0.85 and the T1 of arterial blood, 1650ms. CBF values were scaled by the equilibrium magnetisation of arterial blood estimated from the average proton density within the lateral ventricles [13].

Proton density weighted images were co-registered with the T1-weighted images using boundary-based registration [8] (FSL version, 5.010, <https://fsl.fmrib.ox.ac.uk/fsl/>). The repaired tissue probability maps were aligned with the pCASL images by applying the inverse of these transformations. FLAIR images were co-registered with the pCASL images using FLIRT [9] (FSL version, 5.010, <https://fsl.fmrib.ox.ac.uk/fsl/>) and used to align WMH masks with the pCASL images. CBF voxels were assigned a tissue class (grey matter, normal-appearing white matter, CSF, WMH) based on the maximum tissue likelihood at each voxel. For each patient the distribution of CBF values within the grey and normal-appearing white matter were entered as empirical priors in a Hidden Markov random field model [14] (FSL’s version, 5.010, <https://fsl.fmrib.ox.ac.uk/fsl/>). This technique improves the segmentation of the CBF maps by reducing partial volume effects and tissue classification errors caused by large pCASL voxel sizes. These new tissue segmentations were used to define the grey matter on the CBF maps. For each patient, the median and normalised peak height of CBF values within the grey matter were calculated.

# **References**

1. Cheung CY-L, Hsu W, Lee ML, Wang JJ, Mitchell P, Lau QP, et al. A new method to measure peripheral retinal vascular caliber over an extended area. Microcirculation. 2010;17: 495–503. doi:10.1111/j.1549-8719.2010.00048.x

2. Cheung CY, Tay WT, Mitchell P, Wang JJ, Hsu W, Lee ML, et al. Quantitative and qualitative retinal microvascular characteristics and blood pressure. J Hypertens. 2011;29: 1380–1391. doi:10.1097/HJH.0b013e328347266c

3. Lambert C, Sam Narean J, Benjamin P, Zeestraten E, Barrick TR, Markus HS. Characterising the grey matter correlates of leukoaraiosis in cerebral small vessel disease. NeuroImage: Clinical. 2015;9: 194–205. doi:10.1016/j.nicl.2015.07.002

4. Spilling CA, Jones PW, Dodd JW, Barrick TR. White matter lesions characterise brain involvement in moderate to severe chronic obstructive pulmonary disease, but cerebral atrophy does not. BMC Pulm Med. 2017;17. doi:10.1186/s12890-017-0435-1

5. Dodd JW, Chung AW, van den Broek MD, Barrick TR, Charlton RA, Jones PW. Brain structure and function in chronic obstructive pulmonary disease: a multimodal cranial magnetic resonance imaging study. American Journal of Respiratory and Critical Care Medicine. 2012;186: 240–245. doi:10.1164/rccm.201202-0355OC

6. Avants BB, Tustison NJ, Song G. Advanced normalization tools (ANTS). Insight j. 2009;2: 1–35.

7. Smith SM, Zhang Y, Jenkinson M, Chen J, Matthews PM, Federico A, et al. Accurate, robust, and automated longitudinal and cross-sectional brain change analysis. Neuroimage. 2002;17: 479–489.

8. Greve DN, Fischl B. Accurate and robust brain image alignment using boundary-based registration. Neuroimage. 2009;48: 63–72. doi:10.1016/j.neuroimage.2009.06.060

9. Jenkinson M, Smith S. A global optimisation method for robust affine registration of brain images. Med Image Anal. 2001;5: 143–156.

10. Chappell MA, Groves AR, Whitcher B, Woolrich MW. Variational Bayesian Inference for a Nonlinear Forward Model. IEEE Transactions on Signal Processing. 2009;57: 223–236. doi:10.1109/TSP.2008.2005752

11. Buxton RB, Wong EC, Frank LR. Dynamics of blood flow and oxygenation changes during brain activation: The balloon model. Magn Reson Med. 1998;39: 855–864. doi:10.1002/mrm.1910390602

12. Alsop DC, Detre JA, Golay X, Günther M, Hendrikse J, Hernandez-Garcia L, et al. Recommended implementation of arterial spin-labeled perfusion MRI for clinical applications: A consensus of the ISMRM perfusion study group and the European consortium for ASL in dementia. Magn Reson Med. 2015;73: 102–116. doi:10.1002/mrm.25197

13. Çavuşoğlu M, Pfeuffer J, Uğurbil K, Uludağ K. Comparison of pulsed arterial spin labeling encoding schemes and absolute perfusion quantification. Magnetic Resonance Imaging. 2009;27: 1039–1045. doi:10.1016/j.mri.2009.04.002

14. Zhang Y, Brady M, Smith S. Segmentation of brain MR images through a hidden Markov random field model and the expectation-maximization algorithm. IEEE transactions on medical imaging. 2001;20: 45–57.
