# Supplementary material for: Factors affecting brain structure in smoking-related diseases: Chronic Obstructive Pulmonary Disease (COPD) and coronary artery disease
Source: PLoS One. 2021 Nov 5;16(11):e0259375. doi: 10.1371/journal.pone.0259375 (PMC8570465; doi:10.1371/journal.pone.0259375)
Supplement: S2 Appendix — (DOCX) [file pone.0259375.s002.docx]

# **Supplementary Results**

**Supplementary Table 1: Respiratory function component**

|  | **Component 1**  Respiratory Function |
| --- | --- |
| FEV_1_ % pred. | **0.932** |
| FVC % pred. | **0.901** |
| Oxygen saturations (%) | **0.763** |

Respiratory variable loadings onto principal components extracted using direct oblimin rotation. Highlighted are component loadings >0.5. FEV_1_ = Forced Expiratory Volume in 1 second, FVC = Forced Vital Capacity.

**Supplementary Table 2: Vascular risk component loadings**

|  | **Component 1**  Glucose Dysregulation | **Component 2**  Blood Pressure | **Component 3**  Plasma Lipids |
| --- | --- | --- | --- |
| Systolic blood pressure | 0.016 | **0.869** | -0.029 |
| Diastolic blood pressure | 0.038 | **0.856** | -0.003 |
| HbA1C | **0.843** | -0.100 | 0.257 |
| HOMA-IR | **0.824** | 0.034 | -0.083 |
| BMI (log_10_) | **0.681** | 0.156 | -0.337 |
| Non-HDL | -0.051 | 0.049 | **-0.904** |
| HDL | -0.185 | 0.461 | **0.536** |

Vascular risk variable loadings (pattern matrix) onto principal components extracted using direct oblimin rotation. Highlighted are component loadings >0.5. HDL = High Density Lipoprotein, BMI = Body Mass Index, HbA1C = Haemoglobin A1C, HOMA-IR = Homeostatic Model Assessment of Insulin Resistance.

**Supplementary Table 3: Retinal microvascular pathology component loadings**

|  | **Component 1**  Retinal Vessel Calibre | **Component 2**  Retinal Vessel Tortuosity |
| --- | --- | --- |
| Arteriole calibre | **0.899** | 0.116 |
| Venule calibre | **0.882** | 0.087 |
| Venule branching angle | -0.383 | 0.115 |
| Total fractal dimension (log_10_ reflected data) | 0.139 | **-0.740** |
| Venule tortuosity (log_10_) | 0.124 | **0.752** |
| Arteriole tortuosity (log_10_) | -0.093 | **0.632** |
| Arteriole branching angle (square root reflected data) | -0.057 | -0.375 |

Retinal microvascular pathology variable loadings (pattern matrix) onto principal components extracted using direct oblimin rotation. Highlighted are component loadings >0.5.

**Supplementary Table 4: Brain Tissue Volume component loadings**

|  | **Component 1**  White Matter Microstructural Damage | **Component 2**  Cerebral Atrophy |
| --- | --- | --- |
| FA peak height | **0.926** | -0.162 |
| Median FA | **-0.912** | -0.080 |
| Median MD | **0.891** | 0.124 |
| CSF volume (% TIV) | **-**0.084 | **0.949** |
| Grey Matter volume (% TIV) | -0.056 | **-0.905** |
| Lateral ventricle volume (log_10_ % TIV) | 0.040 | **0.745** |

Brain Tissue Volume variable loadings (pattern matrix) onto principal components extracted using direct oblimin rotation. Highlighted are component loadings >0.5. FA = Fractional anisotropy, MD = Mean diffusivity, CSF = Cerebrospinal fluid, TIV = Total intracranial volume.

**Supplementary Table 5: Correlations between non-brain measures**

| Whole cohort |  | **Correlations** | | | | | | | | | | | | | | | |
| --- | --- | --- | --- | --- | --- | --- | --- | --- | --- | --- | --- | --- | --- | --- | --- | --- | --- |
|  | 1 | 2 | 3 | 4 | 5 | 6 | 7 | 8 | 9 | 10 | 11 | 12 | 13 | 14 | 15 | 16 |  |
| 1. Respiratory Function^†^ | 1.00 |  |  |  |  |  |  |  |  |  |  |  |  |  |  |  |  |
| 1. MRC dyspnoea | -0.60^***^ | 1.00 |  |  |  |  |  |  |  |  |  |  |  |  |  |  |  |
| 1. Pack years (square root) | -0.27^**^ | 0.34^***^ | 1.00 |  |  |  |  |  |  |  |  |  |  |  |  |  |  |
| 1. Plasma Lipids^†^ | -0.23^*^ | 0.25 | 0.05 | 1.00 |  |  |  |  |  |  |  |  |  |  |  |  |  |
| 1. Blood Pressure^†^ | -0.35^***^ | 0.10 | -0.07 | 0.10 | 1.00 |  |  |  |  |  |  |  |  |  |  |  |  |
| 1. Glucose Dysregulation^†^ | -0.06 | 0.09 | 0.08 | -0.22^*^ | -0.12 | 1.00 |  |  |  |  |  |  |  |  |  |  |  |
| 1. Pulse wave velocity (log_10_) | -0.15 | 0.10 | -0.17 | -0.02 | 0.17 | 0.10 | 1.00 |  |  |  |  |  |  |  |  |  |  |
| 1. Troponin T (log_10_) | -0.42^***^ | 0.38^***^ | 0.09 | 0.39^***^ | 0.34^**^ | -0.12 | 0.08 | 1.00 |  |  |  |  |  |  |  |  |  |
| 1. Median CBF | -0.25^*^ | 0.17 | 0.13 | 0.14 | -0.22^*^ | -0.06 | -0.06 | 0.07 | 1.00 |  |  |  |  |  |  |  |  |
| 1. CBF peak height | 0.28^**^ | -0.21^*^ | -0.24^*^ | -0.14 | 0.24^*^ | -0.08 | 0.11 | -0.07 | -0.88^***^ | 1.00 |  |  |  |  |  |  |  |
| 1. Hs-CRP | -0.09 | 0.30^**^ | 0.27^**^ | -0.23^*^ | 0.01 | 0.21 | -0.00 | 0.03 | -0.15 | 0.16 | 1.00 |  |  |  |  |  |  |
| 1. Fibrinogen | -0.10 | 0.18 | 0.15 | 0.04 | 0.05 | 0.20 | 0.10 | -0.02 | -0.11 | 0.10 | 0.42^***^ | 1.00 |  |  |  |  |  |
| 1. Neutrophil count | -0.04 | 0.07 | 0.19 | 0.05 | -0.05 | 0.01 | -0.04 | 0.13 | -0.08 | 0.02 | 0.26^**^ | 0.07 | 1.00 |  |  |  |  |
| 1. Urine ACR (log_10_) | -0.17 | 0.02 | 0.10 | 0.23 | 0.27^*^ | -0.28^*^ | 0.10 | 0.22 | -0.27^*^ | 0.24 | -0.10 | -0.10 | 0.15 | 1.00 |  |  |  |
| 1. Retinal Vessel Calibre^†^ | 0.06 | -0.10 | -0.03 | -0.25^*^ | -0.29^*^ | -0.10 | -0.03 | -0.15 | 0.02 | 0.02 | 0.13 | 0.01 | 0.23^*^ | -0.02 | 1.00 |  |  |
| 1. Retinal Vessel Tortuosity^†^ | -0.21 | -0.00 | -0.13 | -0.04 | -0.16 | 0.11 | 0.05 | -0.11 | 0.11 | -0.07 | 0.13 | 0.14 | 0.09 | 0.06 | -0.06 | 1.00 |  |

Pearson correlations coefficients showing relationships between clinical variables and components. Age and sex have been regressed out of all analyses. Significant values are highlighted in Bold. *p<0.05, **p<0.01, ***p<0.001. ^†^Principal Component. ACR = Albumin to Creatinine Ratio, Pack years = Pack years smoking history, MRC = Medical Research Council, Hs-CRP = High-sensitivity C-Reactive Protein, CBF = Cerebral Blood Flow. Results are not corrected for multiple comparisons.

**Supplementary Table 6**

| **Single variable models (hierarchical linear regression) – Brain Tissue Volume** | | | | | | | | | | | |
| --- | --- | --- | --- | --- | --- | --- | --- | --- | --- | --- | --- |
| **Predictor** |  | **Main Effect**  **(Disease marker)** | | **Main Effect**  **(Group)** | | | **Interaction**  **(Group × Disease marker)** | | **Overall model** | | |
|  | Step | *t* | *p* | | *t* | *p* | *t* | *p* | adj. *r*^2^ | *F* | *p* |
| Respiratory Function^†^ | 1 | -0.784 | 0.435 | |  |  |  |  | -0.004 | 0.614 | 0.435 |
|  | 2 | -1.033 | 0.304 | | -0.703 | 0.484 |  |  | -0.009 | 0.553 | 0.577 |
|  | 3 | -0.704 | 0.483 | | -0.737 | 0.463 | 0.234 | 0.816 | -0.019 | 0.383 | 0.766 |
| MRC dyspnoea | 1 | 0.183 | 0.855 | |  |  |  |  | -0.010 | 0.033 | 0.855 |
|  | 2 | 0.371 | 0.712 | | -0.367 | 0.714 |  |  | -0.019 | 0.084 | 0.919 |
|  | 3 | 0.047 | 0.963 | | -0.275 | 0.784 | 0.147 | 0.884 | -0.030 | 0.063 | 0.979 |
| Pack years (square root) | 1 | 0.112 | 0.911 | |  |  |  |  | -0.010 | 0.013 | 0.911 |
|  | 2 | 0.167 | 0.868 | | -0.215 | 0.830 |  |  | -0.020 | 0.029 | 0.971 |
|  | 3 | 1.146 | 0.255 | | -0.318 | 0.751 | -1.324 | 0.189 | -0.012 | 0.604 | 0.614 |
| Blood Pressure^†^ | 1 | 4.133 | <0.001^***^ | |  |  |  |  | 0.153 | 17.082 | <0.001^***^ |
|  | 2 | 4.399 | <0.001^***^ | | -1.440 | 0.153 |  |  | 0.163 | 9.682 | <0.001^***^ |
|  | 3 | 2.078 | 0.041^*^ | | -1.344 | 0.182 | 0.534 | 0.595 | 0.156 | 6.497 | 0.001^**^ |
| Glucose Dysregulation^†^ | 1 | -1.162 | 0.249 | |  |  |  |  | 0.004 | 1.349 | 0.249 |
|  | 2 | -1.167 | 0.246 | | -0.189 | 0.851 |  |  | -0.007 | 0.685 | 0.507 |
|  | 3 | -0.205 | 0.838 | | -0.204 | 0.839 | -0.644 | 0.521 | -0.014 | 0.592 | 0.622 |
| Pulse wave velocity (log_10_) | 1 | 0.915 | 0.363 | |  |  |  |  | -0.002 | 0.837 | 0.363 |
|  | 2 | 0.921 | 0.359 | | -0.227 | 0.821 |  |  | -0.012 | 0.440 | 0.645 |
|  | 3 | 0.387 | 0.700 | | -0.227 | 0.821 | 0.323 | 0.747 | -0.021 | 0.325 | 0.807 |
| Troponin T (log_10_) | 1 | 1.754 | 0.083 | |  |  |  |  | 0.023 | 3.076 | 0.083 |
|  | 2 | 1.852 | 0.067 | | -0.616 | 0.540 |  |  | 0.016 | 1.717 | 0.186 |
|  | 3 | 1.514 | 0.134 | | -0.736 | 0.464 | -0.628 | 0.532 | 0.009 | 2.268 | 0.290 |
| Median CBF | 1 | -4.795 | <0.001^***^ | |  |  |  |  | 0.191 | 22.989 | <0.001^***^ |
|  | 2 | -4.827 | <0.001^***^ | | 0.834 | 0.406 |  |  | 0.189 | 11.804 | <0.001^***^ |
|  | 3 | -3.507 | 0.001^**^ | | 0.840 | 0.403 | -0.278 | 0.782 | 0.180 | 7.816 | <0.001^***^ |
| CBF peak height | 1 | 3.736 | <0.001^***^ | |  |  |  |  | 0.122 | 13.961 | <0.001^***^ |
|  | 2 | 3.725 | <0.001^***^ | | 0.549 | 0.584 |  |  | 0.116 | 7.078 | 0.001^**^ |
|  | 3 | 2.483 | 0.015^*^ | | 0.545 | 0.587 | 0.338 | 0.736 | 0.107 | 4.711 | 0.004^**^ |
| Hs-CRP (log_10_) | 1 | -0.939 | 0.350 | |  |  |  |  | -0.001 | 0.881 | 0.350 |
|  | 2 | -0.924 | 0.358 | | -0.224 | 0.824 |  |  | -0.011 | 0.461 | 0.632 |
|  | 3 | -0.718 | 0.474 | | -0.223 | 0.824 | 0.114 | 0.910 | -0.022 | 0.308 | 0.819 |
| Fibrinogen (log_10_) | 1 | -0.438 | 0.629 | |  |  |  |  | -0.008 | 0.234 | 0.629 |
|  | 2 | -0.457 | 0.649 | | -0.104 | 0.917 |  |  | -0.018 | 0.121 | 0.886 |
|  | 3 | 1.986 | 0.050^*^ | | -0.326 | 0.746 | 2.763 | 0.007^**^ | 0.048 | 2.632 | 0.055 |
| Neutrophil count (log_10_) | 1 | -0.189 | 0.851 | |  |  |  |  | -0.010 | 0.036 | 0.851 |
|  | 2 | -0.187 | 0.852 | | -0.175 | 0.861 |  |  | -0.020 | 0.033 | 0.968 |
|  | 3 | -0.906 | 0.367 | | -0.183 | 0.855 | 1.008 | 0.316 | -0.020 | 0.361 | 0.781 |
| Urine ACR (log_10_) | 1 | 0.954 | 0.343 | |  |  |  |  | -0.001 | 0.910 | 0.343 |
|  | 2 | 0.946 | 0.347 | | -0.119 | 0.906 |  |  | -0.016 | 0.456 | 0.636 |
|  | 3 | 1.249 | 0.216 | | -0.101 | 0.920 | -0.825 | 0.412 | -0.021 | 0.529 | 0.664 |
| Retinal Vessel Calibre^†^ | 1 | 0.352 | 0.726 | |  |  |  |  | -0.011 | 0.124 | 0.726 |
|  | 2 | 0.225 | 0.822 | | -0.479 | 0.633 |  |  | -0.021 | 0.176 | 0.839 |
|  | 3 | -0.358 | 0.721 | | -0.440 | 0.661 | 0.797 | 0.428 | -0.025 | 0.329 | 0.804 |
| Retinal Vessel Tortuosity^†^ | 1 | -1.114 | 0.269 | |  |  |  |  | 0.003 | 1.241 | 0.269 |
|  | 2 | -1.081 | 0.283 | | -0.496 | 0.621 |  |  | -0.006 | 0.737 | 0.482 |
|  | 3 | -0.833 | 0.407 | | -0.493 | 0.623 | 0.114 | 0.909 | -0.019 | 0.490 | 0.690 |

Three-step hierarchical linear model showing the simple relationship between each disease marker and Brain Tissue Volume. Step 1 predictors: disease marker only; Step 2 predictors: disease marker, group; Step 3 predictors: disease marker, group, group × disease marker interaction. Reported are the *t*-statistics (*t*) and *p*-values for each main effect and interaction, as well as the regression coefficient (*r*^2^), adjusted regression coefficient (adj *r*^2^), *F*-statistics (*F*) and *p*-values (*p*) for the overall models. The COPD>CAD contrast for the main effect of group and the group × disease marker interaction is shown. All models included a constant term (not shown). All variables were adjusted for age and sex. ^†^Principal component, **p*<0.05, ***p*<0.01, ****p*<0.001, ACR = Albumin to Creatinine Ratio, CBF = Cerebral Blood Flow, CRP = high-sensitivity C-Reactive Protein, MRC = Medical Research Council.

**Supplementary Table 7**

| **Single variable models (hierarchical linear regression) – WMH volume** | | | | | | | | | | |
| --- | --- | --- | --- | --- | --- | --- | --- | --- | --- | --- |
| **Predictor** |  | **Main Effect**  **(Disease marker)** | | **Main Effect**  **(Group)** | | **Interaction**  **(Group × Disease marker)** | | **Overall model** | | |
|  | Step | *t* | *p* | *t* | *p* | *t* | *p* | adj. *r*^2^ | *F* | *p* |
| Respiratory Function^†^ | 1 | -2.559 | 0.012^*^ |  |  |  |  | 0.054 | 6.550 | 0.012^*^ |
|  | 2 | -2.501 | 0.014^*^ | -0.616 | 0.539 |  |  | 0.048 | 3.444 | 0.036^*^ |
|  | 3 | -1.466 | 0.146 | -0.676 | 0.501 | 0.292 | 0.771 | 0.039 | 2.302 | 0.082 |
| MRC dyspnoea | 1 | 2.430 | 0.017^*^ |  |  |  |  | 0.048 | 5.906 | 0.017^*^ |
|  | 2 | 2.345 | 0.021^*^ | -0.714 | 0.477 |  |  | 0.043 | 3.193 | 0.045^*^ |
|  | 3 | 0.596 | 0.533 | -0.413 | 0.681 | 0.588 | 0.558 | 0.036 | 2.230 | 0.090 |
| Pack years (square root) | 1 | 1.985 | 0.050^*^ |  |  |  |  | 0.029 | 3.939 | 0.050^*^ |
|  | 2 | 1.787 | 0.077 | 0.396 | 0.693 |  |  | 0.021 | 2.031 | 0.137 |
|  | 3 | 1.042 | 0.300 | 0.398 | 0.691 | 0.069 | 0.945 | 0.010 | 1.342 | 0.266 |
| Blood Pressure^†^ | 1 | 3.034 | 0.003^**^ |  |  |  |  | 0.084 | 9.206 | 0.003^**^ |
|  | 2 | 2.830 | 0.006^**^ | 0.137 | 0.892 |  |  | 0.074 | 4.561 | 0.013^*^ |
|  | 3 | 2.144 | 0.035^*^ | 0.045 | 0.965 | -0.640 | 0.524 | 0.068 | 3.157 | 0.029^*^ |
| Glucose Dysregulation^†^ | 1 | -0.682 | 0.497 |  |  |  |  | -0.006 | 0.466 | 0.497 |
|  | 2 | -0.595 | 0.553 | 0.956 | 0.343 |  |  | -0.007 | 0.689 | 0.505 |
|  | 3 | -0.753 | 0.453 | 0.963 | 0.338 | 0.493 | 0.623 | -0.016 | 0.536 | 0.659 |
| Pulse wave velocity (log_10_) | 1 | -0.577 | 0.565 |  |  |  |  | -0.007 | 0.333 | 0.565 |
|  | 2 | -0.629 | 0.531 | 0.952 | 0.344 |  |  | -0.008 | 0.620 | 0.540 |
|  | 3 | -0.052 | 0.959 | 0.951 | 0.344 | -0.510 | 0.611 | -0.016 | 0.497 | 0.685 |
| Troponin T (log_10_) | 1 | 2.443 | 0.017^*^ |  |  |  |  | 0.052 | 5.967 | 0.017^*^ |
|  | 2 | 2.066 | 0.042^*^ | 0.522 | 0.603 |  |  | 0.044 | 3.096 | 0.050^*^ |
|  | 3 | 2.197 | 0.031^*^ | 0.230 | 0.819 | -1.291 | 0.200 | 0.052 | 2.635 | 0.055 |
| Median CBF | 1 | -1.407 | 0.163 |  |  |  |  | 0.010 | 1.980 | 0.163 |
|  | 2 | -1.664 | 0.099 | 1.106 | 0.272 |  |  | 0.013 | 1.604 | 0.207 |
|  | 3 | -1.579 | 0.118 | 1.125 | 0.264 | 0.604 | 0.548 | 0.006 | 1.183 | 0.321 |
| CBF peak height | 1 | 1.535 | 0.128 |  |  |  |  | 0.014 | 2.356 | 0.128 |
|  | 2 | 1.801 | 0.075 | 1.148 | 0.254 |  |  | 0.018 | 1.841 | 0.164 |
|  | 3 | 1.746 | 0.084 | 1.147 | 0.255 | -0.633 | 0.528 | 0.011 | 1.353 | 0.262 |
| Hs-CRP (log_10_) | 1 | 3.125 | 0.002^**^ |  |  |  |  | 0.084 | 9.765 | 0.002 |
|  | 2 | 3.082 | 0.003^**^ | 0.885 | 0.378 |  |  | 0.082 | 5.263 | 0.007^**^ |
|  | 3 | 2.840 | 0.006^**^ | 0.888 | 0.377 | -0.980 | 0.330 | 0.081 | 3.827 | 0.012^*^ |
| Fibrinogen (log_10_) | 1 | 3.948 | 0.050^*^ |  |  |  |  | 0.029 | 1.987 | 0.050^*^ |
|  | 2 | 1.853 | 0.067 | 0.585 | 0.560 |  |  | 0.023 | 2.132 | 0.124 |
|  | 3 | 2.089 | 0.039^*^ | 0.487 | 0.627 | -1.246 | 0.216 | 0.028 | 1.947 | 0.127 |
| Neutrophil count (log_10_) | 1 | 0.837 | 0.404 |  |  |  |  | -0.003 | 0.701 | 0.404 |
|  | 2 | 0.836 | 0.405 | 0.920 | 0.360 |  |  | -0.005 | 0.773 | 0.465 |
|  | 3 | -1.485 | 0.141 | 0.925 | 0.357 | 2.581 | 0.011^*^ | 0.051 | 2.766 | 0.046^*^ |
| Urine ACR (log_10_) | 1 | 0.781 | 0.438 |  |  |  |  | -0.006 | 0.610 | 0.438 |
|  | 2 | 0.782 | 0.437 | 0.645 | 0.521 |  |  | -0.014 | 0.511 | 0.602 |
|  | 3 | 0.397 | 0.693 | 0.636 | 0.528 | 0.199 | 0.843 | -0.029 | 0.349 | 0.790 |
| Retinal Vessel Calibre^†^ | 1 | -0.550 | 0.584 |  |  |  |  | -0.009 | 0.303 | 0.584 |
|  | 2 | -0.393 | 0.695 | 0.575 | 0.567 |  |  | -0.017 | 0.315 | 0.730 |
|  | 3 | -1.120 | 0.266 | 0.634 | 0.528 | 1.246 | 0.217 | -0.010 | 0.729 | 0.538 |
| Retinal Vessel Tortuosity^†^ | 1 | 0.080 | 0.937 |  |  |  |  | -0.012 | 0.006 | 0.937 |
|  | 2 | 0.043 | 0.965 | 0.686 | 0.495 |  |  | -0.019 | 0.238 | 0.789 |
|  | 3 | 0.842 | 0.403 | 0.690 | 0.492 | -1.135 | 0.260 | -0.015 | 0.589 | 0.624 |

Three-step hierarchical linear model showing the simple relationship between each disease marker and WMH volume. Step 1 predictors: disease marker only; Step 2 predictors: disease marker, group; Step 3 predictors: disease marker, group, group × disease marker interaction. Reported are the *t*-statistics (*t*) and *p*-values for each main effect and interaction, as well as the regression coefficient (*r*^2^), adjusted regression coefficient (adj *r*^2^), *F*-statistics (*F*) and *p*-values (*p*) for the overall models. The COPD>CAD contrast for the main effect of group and the group × disease marker interaction is shown. All models included a constant term (not shown). All variables were adjusted for age and sex. ^†^Principal component, **p*<0.05, ***p*<0.01, ****p*<0.001. ACR = Albumin to Creatinine Ratio, CBF = Cerebral Blood Flow, CRP = high-sensitivity C-Reactive Protein, MRC = Medical Research Council, WMH = White Matter Hyperintense Lesions.

**Supplementary Table 8**

| **Single variable models (hierarchical linear regression) – White Matter Microstructure** | | | | | | | | | | |
| --- | --- | --- | --- | --- | --- | --- | --- | --- | --- | --- |
| **Predictor** |  | **Main Effect**  **(Disease marker)** | | **Main Effect**  **(Group)** | | **Interaction**  **(Group × Disease marker)** | | **Overall model** | | |
|  | Step | *t* | *p* | *t* | *p* | *t* | *p* | adj. *r*^2^ | *F* | *p* |
| Respiratory Function^†^ | 1 | -6.335 | <0.001^***^ |  |  |  |  | 0.287 | 40.126 | <0.001^***^ |
|  | 2 | -4.133 | <0.001^***^ | 2.855 | 0.005^**^ |  |  | 0.337 | 25.634 | <0.001^***^ |
|  | 3 | -1.797 | 0.076 | 2.757 | 0.007^**^ | -0.234 | 0.816 | 0.330 | 16.938 | <0.001^***^ |
| MRC dyspnoea | 1 | 5.084 | <0.001^***^ |  |  |  |  | 0.202 | 25.847 | <0.001^***^ |
|  | 2 | 2.290 | 0.024^*^ | 2.999 | 0.003^**^ |  |  | 0.263 | 18.484 | <0.001^***^ |
|  | 3 | 2.138 | 0.035^*^ | 2.266 | 0.026^*^ | -1.188 | 0.238 | 0.266 | 12.846 | <0.001^***^ |
| Pack years (square root) | 1 | 1.974 | 0.051 |  |  |  |  | 0.029 | 3.895 | 0.051 |
|  | 2 | 0.705 | 0.482 | 5.083 | <0.001^***^ |  |  | 0.227 | 15.366 | <0.001^***^ |
|  | 3 | 0.481 | 0.631 | 5.037 | <0.001^***^ | -0.061 | 0.951 | 0.219 | 10.139 | <0.001^***^ |
| Blood Pressure^†^ | 1 | 3.984 | <0.001^***^ |  |  |  |  | 0.143 | 15.875 | <0.001^***^ |
|  | 2 | 2.895 | 0.005^**^ | 3.883 | <0.001^***^ |  |  | 0.261 | 16.745 | <0.001^***^ |
|  | 3 | 1.180 | 0.241 | 3.911 | <0.001^***^ | 0.582 | 0.562 | 0.256 | 11.191 | <0.001^***^ |
| Glucose Dysregulation^†^ | 1 | -0.413 | 0.681 |  |  |  |  | -0.009 | 0.170 | 0.681 |
|  | 2 | -0.045 | 0.964 | 4.764 | <0.001^***^ |  |  | 0.190 | 11.452 | <0.001^***^ |
|  | 3 | 0.402 | 0.689 | 4.730 | <0.001^***^ | -0.544 | 0.588 | 0.184 | 7.672 | <0.001^***^ |
| Pulse wave velocity (log_10_) | 1 | -0.259 | 0.796 |  |  |  |  | -0.010 | 0.067 | 0.796 |
|  | 2 | -0.600 | 0.550 | 5.519 | <0.001^***^ |  |  | 0.226 | 15.276 | <0.001^***^ |
|  | 3 | -1.142 | 0.256 | 5.515 | <0.001^***^ | 1.001 | 0.319 | 0.226 | 10.518 | <0.001^***^ |
| Troponin T (log_10_) | 1 | 4.796 | <0.001^***^ |  |  |  |  | 0.196 | 4.796 | <0.001^***^ |
|  | 2 | 3.353 | 0.001^**^ | 3.886 | <0.001^***^ |  |  | 0.306 | 20.876 | <0.001^***^ |
|  | 3 | 1.743 | 0.085 | 3.779 | <0.001^***^ | 0.039 | 0.969 | 0.298 | 13.760 | <0.001^***^ |
| Median CBF | 1 | 1.059 | 0.292 |  |  |  |  | 0.001 | 1.121 | 0.292 |
|  | 2 | -0.228 | 0.820 | 4.793 | <0.001^***^ |  |  | 0.194 | 12.179 | <0.001^***^ |
|  | 3 | -0.440 | 0.661 | 4.782 | <0.001^***^ | 0.392 | 0.696 | 0.186 | 8.095 | <0.001^***^ |
| CBF peak height | 1 | -0.647 | 0.519 |  |  |  |  | -0.006 | 0.418 | 0.519 |
|  | 2 | 0.712 | 0.478 | 4.941 | <0.001^***^ |  |  | 0.198 | 12.467 | <0.001^***^ |
|  | 3 | 0.542 | 0.589 | 4.914 | <0.001^***^ | -0.035 | 0.973 | 0.189 | 8.221 | <0.001^***^ |
| Hs-CRP (log_10_) | 1 | 2.142 | 0.035^*^ |  |  |  |  | 0.036 | 2.142 | 0.035^*^ |
|  | 2 | 2.221 | 0.029^*^ | 5.552 | <0.001^***^ |  |  | 0.266 | 18.428 | <0.001^***^ |
|  | 3 | 2.551 | 0.012^*^ | 5.585 | <0.001^***^ | -1.394 | 0.167 | 0.274 | 13.056 | <0.001^***^ |
| Fibrinogen (log_10_) | 1 | 0.885 | 0.378 |  |  |  |  | -0.002 | 0.784 | 0.378 |
|  | 2 | 0.062 | 0.951 | 5.576 | <0.001^***^ |  |  | 0.237 | 16.059 | <0.001^***^ |
|  | 3 | 1.870 | 0.065 | 5.498 | <0.001^***^ | -2.245 | 0.027^*^ | 0.268 | 12.841 | <0.001^***^ |
| Neutrophil count(log_10_) | 1 | -0.924 | 0.358 |  |  |  |  | -0.001 | 0.855 | 0.358 |
|  | 2 | -1.058 | 0.293 | 5.517 | <0.001^***^ |  |  | 0.232 | 15.774 | <0.001^***^ |
|  | 3 | -0.862 | 0.391 | 5.488 | <0.001^***^ | 0.264 | 0.792 | 0.224 | 10.437 | <0.001^***^ |
| Urine ACR (log_10_) | 1 | 0.137 | 0.712 |  |  |  |  | -0.013 | 0.137 | 0.712 |
|  | 2 | 0.442 | 0.660 | 4.103 | <0.001^***^ |  |  | 0.179 | 8.502 | 0.001^**^ |
|  | 3 | 0.167 | 0.868 | 4.068 | <0.001^***^ | 0.192 | 0.848 | 0.167 | 5.599 | 0.002^**^ |
| Retinal Vessel Calibre^†^ | 1 | -0.648 | 0.519 |  |  |  |  | -0.007 | 0.419 | 0.519 |
|  | 2 | 0.580 | 0.564 | 5.460 | <0.001^***^ |  |  | 0.257 | 15.191 | <0.001^***^ |
|  | 3 | 0.221 | 0.826 | 5.439 | <0.001^***^ | 0.321 | 0.749 | 0.249 | 10.048 | <0.001^***^ |
| Retinal Vessel Tortuosity^†^ | 1 | 1.384 | 0.170 |  |  |  |  | 0.011 | 1.916 | 0.170 |
|  | 2 | 1.324 | 0.189 | 5.453 | <0.001^***^ |  |  | 0.270 | 16.164 | <0.001^***^ |
|  | 3 | 0.579 | 0.564 | 5.425 | <0.001^***^ | 0.479 | 0.633 | 0.263 | 10.748 | <0.001^***^ |

Three-step hierarchical linear model showing the simple relationship between each disease marker and White Matter Microstructure. Step 1 predictors: disease marker only; Step 2 predictors: disease marker, group; Step 3 predictors: disease marker, group, group × disease marker interaction. Reported are the *t*-statistics (*t*) and *p*-values for each main effect and interaction, as well as the regression coefficient (*r*^2^), adjusted regression coefficient (adj *r*^2^), *F*-statistics (*F*) and *p*-values (*p*) for the overall models. The COPD>CAD contrast for the main effect of group and the group × disease marker interaction is shown. All models included a constant term (not shown). All variables were adjusted for age and sex. ^†^Principal component, **p*<0.05, ***p*<0.01, ****p*<0.001. ACR = Albumin to Creatinine Ratio, CBF = Cerebral Blood Flow, CRP = high-sensitivity C-Reactive Protein, MRC = Medical Research Council.
